# Age affects the association between socioeconomic status and infertility: a cross-sectional study

**DOI:** 10.1186/s12905-023-02680-x

**Published:** 2023-12-19

**Authors:** Xiting Chen, Jiemei Liang, Qian Yang, Jinfa Huang, Lixin Li, Kaixian Deng

**Affiliations:** Department of Gynecology, Shunde Hospital, Southern Medical University (The First People’s Hospital of Shunde), No.1 Jiazi Road, Lunjiao, Shunde District, Foshan City, 528308 Guangdong Province China

**Keywords:** Infertility, Socioeconomic status, Poverty income ratio, Age.

## Abstract

**Background:**

Previous studies have shown the interaction between age and socioeconomic status (SES) on the risk of infertility in the UK, but the association is still unclear in the United States. Therefore, the present study investigated the effect of age on the relationship between SES and the risk of infertility in American women.

**Methods:**

The study included adults who participated in the National Health and Nutrition Examination Survey (NHANES) from 2013 to 2018. The poverty income ratio (PIR) was used to represent the SES of the population. With participants stratified according to age category (< 35 years; ≥ 35 years), we further assessed differences in the relationship between PIR and infertility risk among participants of different age groups using multivariate logistic regression and interaction tests.

**Results:**

Approximately 3,273 participants were enrolled in the study. There were 399 cases of infertility and 2,874 cases without infertility. In women ≥ 35 years of age, PIR levels were significantly higher in infertile participants than in non-infertile participants, but no such difference was found in those < 35 years of age. The association of PIR with the risk of infertility appeared to differ between age < 35 years and age ≥ 35 years (OR: 0.99, 95%Cl: 0.86–1.13 vs. OR: 1.24, 95%Cl: 1.12–1.39) in a fully adjusted model. Furthermore, an interaction between age and PIR increased the risk of infertility (p-value for interaction < 0.001).

**Conclusion:**

Our study found that age may influence the association between PIR and infertility. It is imperative to perform further studies to provide more evidence.

**Supplementary Information:**

The online version contains supplementary material available at 10.1186/s12905-023-02680-x.

## Background

Social factors play a crucial role in regulating human physical and mental health [1]. The World Health Organization (WHO) confirmed the significance of socioeconomic status (SES) as an important measure of social factors and its significant effect on mortality and morbidity [2]. Several studies have shown that SES is inversely associated with the risk of cardiovascular disease [3], diabetes [4], depression [5], and obesity [4]. In reproductive health, SES has been reported to be directly related to age at menarche, suggesting a lower age at menarche in adolescents with parents of low SES [6]. Furthermore, studies have found an inverse association between SES and adverse pregnancy outcomes, such as stillbirth and low birth weight [7, 8].

Infertility is recognized by the WHO as a global public health problem and is typically defined as the inability to conceive after 1 year of regular unprotected intercourse [9], which has affected 48.5 million couples worldwide [10]. Infertility not only affects social and population development but also contributes to mental health disorders in individuals [11, 12]. SES may affect fertility in ways, including changing women’s working and living conditions and access to better material resources and services. The relationship between SES and infertility has been studied in the US, but the results have been inconsistent. An American cross-sectional study reported that SES and its downstream-related factors were not associated with infertility [13]. The National Survey of Family Growth of the US from 1982 to 2010 showed no significant correlation between the percentage of the poverty level and infertility risk [14]. However, the results from 1995 to 2019 showed that the infertility probability of middle-income groups was significantly lower than that of low-income and high-income groups [15]. Thus, we suspect there may be potential confounding factors that have not been fully considered to account for this discrepancy.

Age is currently one of the most important indicators affecting fertility. A cohort study on the UK Primary Care Database [16] confirmed the interaction between age and SES on the risk of infertility. Research has shown that women from more socioeconomically deprived groups have recorded higher rates of fertility problems than other women before the age of 25 years. After the age of 25 years, the results have been reversed. In the US, however, no studies have evaluated whether there are differences in the risk of infertility in different SES across different age groups.

Therefore, we evaluated the effect of age on the relationship between SES and infertility risk in the US community using the National Health and Nutrition Examination Survey (NHANES) database from 2013 to 2018.

## Methods

### Data sources

NHANES is an annual cross-sectional survey, which assesses the health and nutritional status of the US population through stratified, multistage cluster sampling. The National Center for Health Statistics developed the survey, and its research ethics review board approved it. Written informed consent was provided by all participants. Information on infertility is only available in NHANES 2013–2020, but because of incomplete data collection during 2019–2020 during the COVID-19 pandemic, we only included NHANES data from 2013 to 2018.

### Design and participants

We combined three 2-year NHANES cycles from 2013 to 2018 to increase the sample size. A total of 29,400 individuals completed survey data during the three survey periods. We excluded male participants (n = 14,452) or those who were < 20 or > 50 years old (n = 10,390). Other exclusion criteria were as follows: (1) missing data for PIR and infertility (n = 1,020), (2) participants with hysterectomy or bilateral ovary removal (n = 265). Consequently, 3,273 participants were included in this study.

### Diagnosis of PIR and infertility

Self-reported infertility was based on participants’ responses to the question, “Have you ever attempted to become pregnant over a period of at least a year without becoming pregnant?”. People who answered “yes” were considered infertile, while those who answered “no” were considered “fertile”.

PIR (poverty income ratio) is calculated by dividing annual household income by the appropriate poverty line for a household set by the US Census Bureau for a given year. The PIR is a useful indicator of SES. Because it is not affected by annual inflation and changes in family size, PIR is comparable across surveys and widely used in multiple NHANES studies [17–19]. A PIR of 1 represents the official federal poverty level, while a PIR of 2 is roughly the median of PIR values from the overall population. Therefore, those with a PIR less than 2 are classified as low SES groups, while those with a PIR greater than or equal to 2 are classified as high SES groups.

### Covariates

Based on previously published literature and clinical significance, we included the following covariates: age, race, marital status, education, health insurance, previous pregnancy, body mass index (BMI), general health condition, pelvic inflammatory disease, smoking, diabetes, hypertension, cancer, coronary heart disease, stroke, and vigorous and moderate recreational activities [20–24]. Multivariable adjustments were made for the variables with a p-value of < 0.10 in the univariate analysis or with the matched odds ratio would change by at least 10%.

A five-group classification of race was made based on the NHANES questionnaire: Mexican American, non-Hispanic Asian, non-Hispanic White, non-Hispanic Black, and other races. Three educational levels (below high school, high school, and above high school) were used. Marital status was divided into two categories (living alone; married or living with a partner). Health insurance was defined as being covered by health insurance or some other kind of health care plan. General health was determined by participants’ questionnaire responses as excellent, very good, good, fair, and poor. Previous pregnancy status was based on survey questions to determine whether you have been pregnant (yes, no). The definitions of hypertension and diabetes were provided by a doctor or other trained health professional. The smoking status was based on self-reports of whether smoked at least 100 cigarettes.

### Statistical analysis

All statistical analyses were performed with the statistical software Free Statistics software version 1.7 [25]. Continuous variables are expressed as mean ± standard deviation, and category variables are expressed as frequency and percentage. We compared quantitative data with the t-test and qualitative data with chi-square and Fisher’s exact tests. Imputation methods were not used because of the small percentage of missing data for all variables in this study (missing rates ranged from 0 to 0.76%).

Univariate and stratified multivariate logistic regression was used to explore the association between PIR and infertility. We used the variance inflation factor (VIF) to test whether there was a multicollinearity relationship between variables. If the VIF was greater than or equal to 5, it indicated the existence of multicollinearity. To assess the effect of age on the relationship between SES and infertility risk, we divided the participants into two groups (< 35 years of age and ≥ 35 years of age). Model 1 is a model without adjustment for any covariates. In Model 2, important sociodemographic factors, including age, race, marriage, education, and health insurance, were considered. Subsequently, Model 3 was formed based on Model 2 by adjusting for BMI and previous pregnancy status. Considering the physical condition and activity of the participants, we further adjusted the general physical condition, smoking, pelvic inflammatory disease, diabetes, hypertension, coronary heart disease, cancer, and vigorous and moderate recreational activities in Model 4 and Model 5. The likelihood ratio test was used to examine the interaction between age, SES, and infertility.

Sensitivity analyses were conducted to assess the robustness of our findings. First, we conducted a sensitivity analysis based on education, another crucial indicator of SES. Second, accounting for the important influence of age on SES and infertility risk, we further subdivided age into six groups with cut-off values of 25, 30, 35, 40, and 45 so that we could better observe the differences in the relationship between SES and infertility risk in different age groups. We presented odds ratio (OR) and 95% confidence interval (CI) with a statistical significance level of 0.05 (two-sided).

## Results

### Characteristics of the participants

**Fig. 1:**
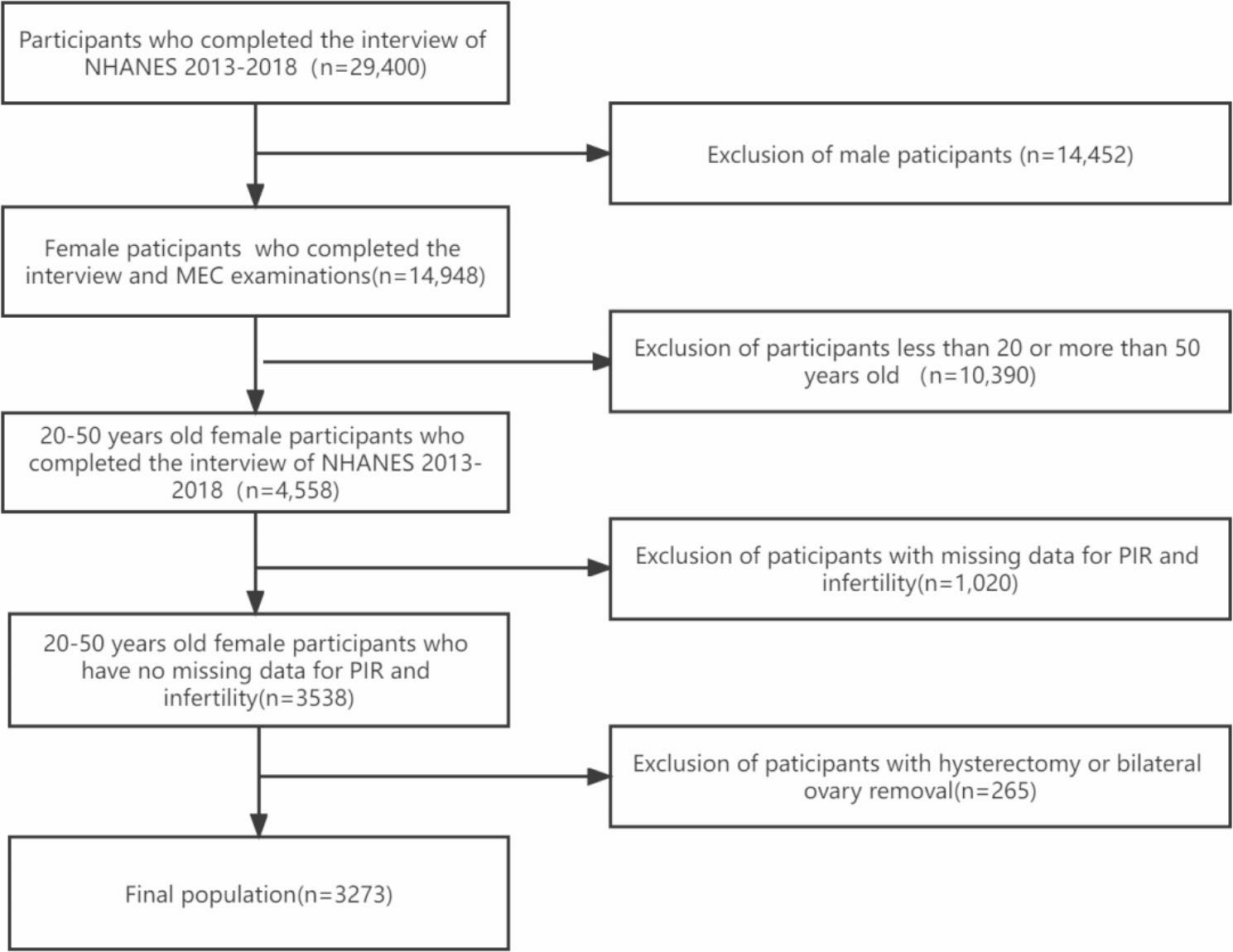
The flow chart of the study

The present study included 29,400 participants who completed interviews. After excluding males (n = 14,452) and participants younger than 20 or older than 50 years old (n = 10,390), we identified 4,558 females aged 20–50 years; 1,020 were excluded because data on PIR and infertility were missing. Besides, participants with hysterectomy or bilateral ovary removal (n = 265) were excluded, and a final analysis of 3,273 participants was conducted. Of the final included population, 399 (12.2%) participants were infertile, and 2,874 (87.8%) were not. The inclusion and exclusion criteria are illustrated by a flow chart in Fig. 1. The number of missing data for all variables in this study ranged from 0 to 25, and the missing rate ranged from 0 to 0.76%.

Table 1 displays the characteristics of this study population with PIR < 2 and PIR ≥ 2. The average age of the 3,273 participants was 34.6 ± 8.8 years old, of whom 1,654 had a PIR < 2 and 1,619 had a PIR ≥ 2. The risk of infertility was higher among participants with PIR ≥ 2 (13.5%) than those with PIR < 2 (10.9%) (p = 0.031). Compared with participants with PIR < 2 (mean age 33.9 ± 8.8 years), those with PIR ≥ 2 were older (mean age 35.3 ± 8.7 years), better educated, and had a lower BMI. A greater proportion of participants with PIR ≥ 2 were married or lived with a partner. Besides, participants with PIR ≥ 2 were more likely to have health insurance and a healthy lifestyle that included moderate or vigorous recreational activities and smoking fewer than 100 cigarettes. We also found that participants with PIR < 2 had worse general health conditions and were more likely to have hypertension, diabetes, and pelvic inflammatory disease.

**Table 1:** Baseline Characteristics of All Participants According to PIR in Our Study

Covariates	Total (n = 3,273)	PIR<2 (n = 1,654)	PIR ≥ 2 (n = 1,619)	P-value
Age, mean ± SD (years)	34.6 ± 8.8	33.9 ± 8.8	35.3 ± 8.7	< 0.001
Race/Ethnicity, n (%)				< 0.001
Mexican American	534 (16.3)	369 (22.3)	165 (10.2)	
Other Hispanic	320 ( 9.8)	174 (10.5)	146 (9)	
Non-Hispanic White	1125 (34.4)	482 (29.1)	643 (39.7)	
Non-Hispanic Black	706 (21.6)	418 (25.3)	288 (17.8)	
Other Race	588 (18.0)	211 (12.8)	377 (23.3)	
Marital status, n (%)				< 0.001
Married or Living with partner	1918 (58.6)	847 (51.2)	1071 (66.2)	
Live alone	1354 (41.4)	806 (48.8)	548 (33.8)	
Education, n (%)				< 0.001
Less than high school	484 (14.8)	407 (24.6)	77 (4.8)	
High school	636 (19.4)	438 (26.5)	198 (12.2)	
More than high school	2151 (65.8)	807 (48.8)	1344 (83)	
Health insurance, n (%)				< 0.001
No	712 (21.8)	569 (34.4)	143 (8.8)	
Yes	2558 (78.2)	1083 (65.6)	1475 (91.2)	
Ever been pregnant, n (%)				< 0.001
No	821 (25.1)	299 (18.1)	522 (32.3)	
Yes	2451 (74.9)	1355 (81.9)	1096 (67.7)	
BMI (kg/m2),Mean ± SD	29.8 ± 8.5	30.8 ± 8.8	28.9 ± 8.1	< 0.001
General health condition, n (%)				< 0.001
Excellent	305 ( 9.3)	122 (7.4)	183 (11.3)	
Very good	957 (29.2)	321 (19.4)	636 (39.3)	
Good	1339 (40.9)	727 (44)	612 (37.8)	
Fair	593 (18.1)	422 (25.5)	171 (10.6)	
Poor	79 ( 2.4)	62 (3.7)	17 (1.1)	
Smoking, n (%)				< 0.001
No	2290 (70.0)	1071 (64.8)	1219 (75.3)	
Yes	981 (30.0)	581 (35.2)	400 (24.7)	
Pelvic inflammatory disease, n (%)				< 0.001
No	3090 (95.0)	1538 (93.6)	1552 (96.4)	
Yes	163 ( 5.0)	105 (6.4)	58 (3.6)	
Diabetes Mellitus, n (%)				0.007
No	3110 (95.0)	1555 (94)	1555 (96.1)	
Yes	162 ( 5.0)	99 (6)	63 (3.9)	
Hypertension, n (%)				< 0.001
No	2731 (83.5)	1341 (81.2)	1390 (85.9)	
Yes	540 (16.5)	311 (18.8)	229 (14.1)	
Coronary heart disease, n (%)				0.125
No	3266 (99.8)	1648 (99.6)	1618 (99.9)	
Yes	7 ( 0.2)	6 (0.4)	1 (0.1)	
Cancer/Malignancy, n (%)				0.614
No	3174 (97.0)	1601 (96.8)	1573 (97.2)	
Yes	99 ( 3.0)	53 (3.2)	46 (2.8)	
Stroke, n (%)				0.081
No	3240 (99.1)	1632 (98.7)	1608 (99.4)	
Yes	31 ( 0.9)	21 (1.3)	10 (0.6)	
Vigorous recreational activities, n (%)				< 0.001
No	2304 (70.4)	1301 (78.7)	1003 (62)	
Yes	969 (29.6)	353 (21.3)	616 (38)	
Moderate recreational activities, n (%)				< 0.001
No	1768 (54.0)	1041 (62.9)	727 (44.9)	
Yes	1505 (46.0)	613 (37.1)	892 (55.1)	
Infertility, n (%)				0.031
0	2874 (87.8)	1473 (89.1)	1401 (86.5)	
1	399 (12.2)	181 (10.9)	218 (13.5)	

### Distribution of PIR in the Infertility Group by Age

**Fig. 2:**
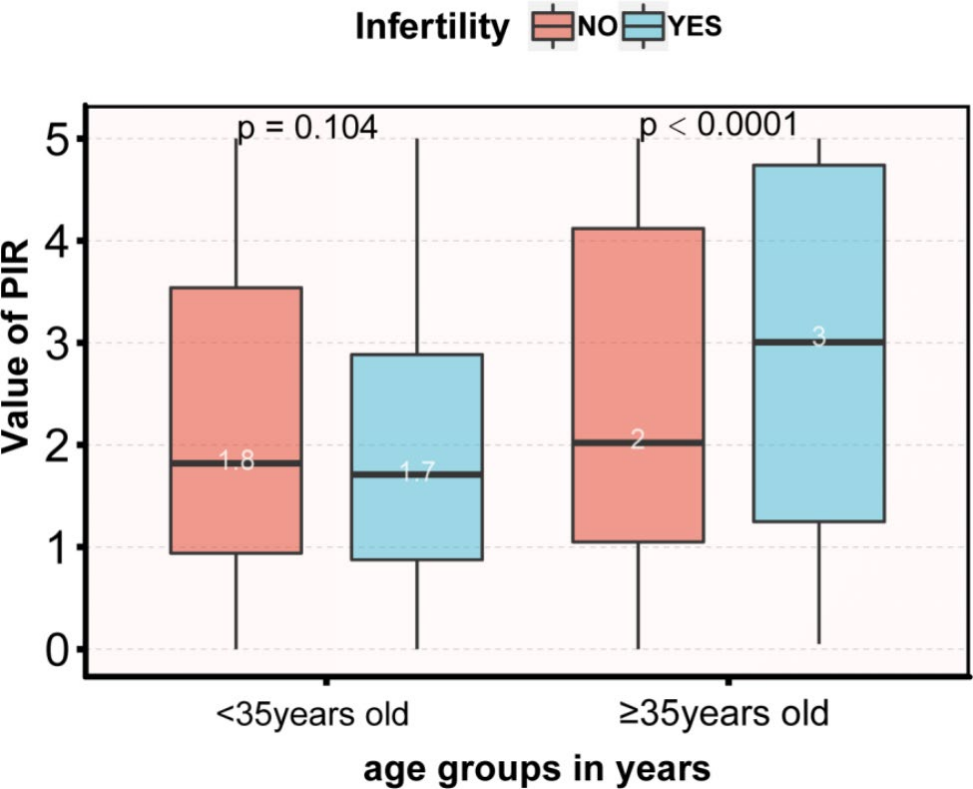
Distribution of PIR values (median and interquartile range) in patients with and without infertility grouped by age

Figure 2 depicts differences in PIR levels between infertile and non-infertile patients among different age groups. The results showed that in women ≥ 35 years of age, infertile participants had significantly higher PIR levels than non-infertile participants (PIR mean 3 vs. 2, p < 0.001). However, the difference was not significant in PIR in women younger than 35 years (PIR mean 1.7 vs. 1.8, p = 0.104).

### Age affects the Association between PIR and Risk of Infertility

Supplementary Table 1 presents the results of univariate analysis, suggesting that age, race, marital status, previous pregnancy, BMI, general physical condition, smoking, pelvic inflammatory disease, diabetes, hypertension, coronary heart disease, cancer, and vigorous recreational activities were associated with infertility. In a fully adjusted model, as PIR increased, the risk of infertility increased significantly in the group aged ≥ 35 years old (OR 1.24, 95% CI, 1.12–1.39, p < 0.001), while the effect with age < 35 years old was not significant (OR 0.99, 95% CI, 0.86–1.13, p = 0.839). When the PIR was converted to a categorical variable with a cut-off value of 2, the results showed that in women aged ≥ 35 years, the OR for the high-SES group was 1.75 (95% CI 1.24–2.47, p = 0.001) compared with the reference group. Meanwhile, at age < 35 years, no significant relationship was found between PIR and infertility risk (OR 1.08, 95% CI 0.71–1.64, p = 0.728). This interaction was also observed in four other models (Table 2).

**Table 2:** Interactive effect of age and PIR on infertility (All participants)

Mode	Variable	<35years old ( n = 1,644 )		≥ 35years old ( n = 1,629 )		P for interaction
		OR (95% CI)	P-value	OR (95% CI)	P-value	
Model l	PIR	0.9 (0.8 ~ 1)	0.055	1.18 (1.09 ~ 1.28)	< 0.001	< 0.001
	PIR<2	1(Ref)		1(Ref)		
	PIR ≥ 2	0.79 (0.56 ~ 1.12)	0.185	1.63 (1.23 ~ 2.14)	0.001	0.001
Model 2	PIR	0.94 (0.82 ~ 1.06)	0.313	1.13 (1.03 ~ 1.25)	0.014	< 0.001
	PIR<2	1(Ref)		1(Ref)		
	PIR ≥ 2	0.95 (0.64 ~ 1.41)	0.799	1.38 (1 ~ 1.9)	0.047	0.004
Model 3	PIR	0.99 (0.87 ~ 1.13)	0.9	1.17 (1.06 ~ 1.29)	0.003	< 0.001
	PIR<2	1(Ref)		1(Ref)		
	PIR ≥ 2	1.09 (0.73 ~ 1.63)	0.685	1.49 (1.07 ~ 2.06)	0.017	0.004
Model 4	PIR	0.99(0.86 ~ 1.13)	0.834	1.22 (1.1 ~ 1.36)	< 0.001	< 0.001
	PIR<2	1(Ref)		1(Ref)		
	PIR ≥ 2	1.09 (0.71 ~ 1.65)	0.702	1.67 (1.19 ~ 2.35)	0.003	0.002
Model 5	PIR	0.99(0.86 ~ 1.13)	0.839	1.24 (1.12 ~ 1.39)	< 0.001	< 0.001
	PIR<2	1(Ref)		1(Ref)		
	PIR ≥ 2	1.08 (0.71 ~ 1.64)	0.728	1.75 (1.24 ~ 2.47)	0.001	0.002

Adjusted covariates:

Model 1: Crude model

Model 2: age, race, marital status, education, health insurance;

Model 3; Model 2 plus BMI, previous pregnancy;

Model 4: Model 3 plus general health condition, smoking, pelvic inflammatory disease, hypertension, diabetes mellitus, coronary heart disease, cancer;

Model 5: Model 4 plus vigorous recreational activities, moderate recreational activities

We also performed several sensitivity analyses on the results. When SES was represented by education, the results remained robust after adjusting for all covariates (Table 3). Table 4 presents the effect of additional age categories on the relationship between PIR and infertility risk. PIR was not significantly associated with the risk of infertility before the age of 35 years. This reversal occurred between the ages of 35 and 45 years, with a higher risk of infertility in women of higher PIR (OR 1.32, 95% CI 1.08–1.62 at ages 35–40 years; OR 1.53, 95% CI 1.25–1.87 at ages 40–45 years). After the age of 45 years, infertility risk did not vary by socioeconomic group.

**Table 3:** Sensitivity Analyses (SES represented by education)

Variable	<35years old ( n = 1,644 )		≥ 35years old ( n = 1,629 )		P for interaction
	OR(95% CI)	P-value	OR(95% CI)	P-value	
SES represented by education					0.024
Less than high school	1(Ref)		1(Ref)		
High school	1.03 (0.57 ~ 1.85)	0.927	1.22(0.72 ~ 2.07)	0.467	
More than high school	0.97 (0.56 ~ 1.67)	0.918	1.54(0.97 ~ 2.44)	0.067	
P for trend	0.98 (0.75 ~ 1.27)	0.873	1.25 (1 ~ 1.55)	0.048	

Adjusted for age, race, marital status, health insurance, BMI, previous pregnancy, general health condition, smoking, pelvic inflammatory disease, hypertension, diabetes mellitus, coronary heart disease, cancer, vigorous recreational activities, moderate recreational activities

**Table 4:** Sensitivity Analyses (Interactive effect of age (additional age categories) and PIR on infertility)

additional age categories	OR (95% CI)	P-value	P for interaction
20–25	0.97 (0.67 ~ 1.42)	0.889	< 0.001
25–30	0.95 (0.74 ~ 1.22)	0.673	
30–35	0.97 (0.78 ~ 1.21)	0.790	
35–40	1.32 (1.08 ~ 1.62)	0.007	
40–45	1.53 (1.25 ~ 1.87)	< 0.001	
45–50	1.02 (0.84 ~ 1.24)	0.828	

Adjusted for age, race, marital status, education, health insurance, BMI, previous pregnancy, general health condition, smoking, pelvic inflammatory disease, hypertension, diabetes mellitus, coronary heart disease, cancer, vigorous recreational activities, moderate recreational activities

## Discussion

Socioeconomic status (SES) is a hierarchical social classification that is influenced by a variety of factors. Currently, the poverty-to-income ratio (PIR) reflects the annual family income relative to the federal poverty and is one of the best measures of SES. Our results demonstrated that in women ≥ 35 years of age, infertile participants had significantly higher PIR levels than non-infertile participants, and PIR was positively associated with the risk of infertility. However, no such phenomenon was found in those < 35 years of age. Age and PIR had an interactive effect on the risk of infertility.

The relationship between SES and infertility has been studied in different countries. A 2011 UK cross-sectional study of 7,702 women showed that women with higher SES reported higher rates of fertility problems [26]. A subsequent 2016 UK study of 8,869 women aged 16–74 years yielded similar results [27]. However, a Portuguese study found no significant differences in income or occupation level among women with and without impaired fertility [28]. A cohort study of primary care databases in the UK suggested that overall infertility rates did not differ by socioeconomic group [16]. These different results may reflect large geographic and national institutional variations in health care.

Furthermore, inconsistency in the study population may be one of the important factors affecting research because some of the aforementioned studies were based on patient samples from medical institutions or health centers. Due to the burden of medical expenses, the study population was mainly composed of women with high SES. Therefore, studies might have ignored many patients with infertility who do not seek medical attention, leading to bias in results. Barut et al. [29] and Surekha et al. [30] have proposed that ovarian reserve indicators, including anti-Mullerian hormone and antral follicle count, positively correlated with SES. Unfortunately, the sample size of the two studies was less than 200, and the age of the study population was 20–35 years old, which could not be extended to the whole population.

As one of the top two countries with increasing wealth inequality, there were only three studies involving SES and infertility in the US. One study included 974 women in the US, suggesting that PIR could not predict infertility [13]. No correlation between poverty and infertility was found in the National Survey of Family Growth from 1982 to 2010 [14]. However, the results from the 1995–2019 National Survey of Family Growth indicated that the middle-income group had a lower probability of infertility than the low-income and high-income groups [15]. These studies provide a fundamental basis for exploring the relationship between SES and infertility. However, none evaluated whether socioeconomic disparities in infertility exist across different age groups. In contrast to previous studies, our study provided further evidence (based on 3,273 women aged 20–50 and 399 events) in the context of an American national community study. With participants stratified according to age category (< 35 years; ≥ 35 years), we found an interactive effect of age on the relationship between PIR and infertility risk through stratified multivariate logistic regression.

Generally, age is currently the best marker for assessing reproductive potential. Previous studies have shown that the peak of female fertility is approximately between the ages of 29 and 30 years in multiparous women and between 27 and 28 years in nulliparous women [31], while the natural fertility and assisted reproductive technology success rates decrease after age 35 [32]. Therefore, 35 years of age was used as the cut-off value for our study population grouping. Age-related infertility is caused by multiple factors. The number of oocytes decreases significantly with the increase in women’s reproductive age [33]. Another important reason may be due to an increase in meiosis errors, leading to the aneuploidy formation of oocytes and embryos, which affects embryo implantation [34]. Furthermore, aging may be associated with increased female reproductive system diseases, including endometrial polyps, endometriosis, uterine fibroids, and tubal disease, leading to impaired female fertility [35]. Declining sexual activity with aging may decrease fertility [33].

Although our study confirmed the interaction of age on SES and infertility, the mechanism remains unclear, which may be related to delayed childbearing. In recent decades, because of women’s increased focus on education and careers and changing attitudes toward personal autonomy and partner’s expectations, there has been an increasing trend toward postponing childbearing [27]. From 1970 to 2002, the proportion of first births among women over 30 increased sixfold [33]. Since 2000, the average age of first-time mothers has risen from 24.9 years to 26.3 years in 2014 in the US [36]. Higher education and higher income are closely related to delayed childbearing [37]. In France, women in high positions over the age of 35 years have experienced the largest increase in fertility [38]. As the reproductive age of women increases, fertility problems become more prominent. Therefore, compared with women with low SES, women with high SES are more likely to delay childbearing until an age when the probability of conception decreases, resulting in higher rates of infertility. Similar phenomena have been found in pregnancy outcomes. The higher incidence of severe maternal morbidity among female doctors may be mediated by advanced maternal age due to delayed childbearing [39], suggesting that age may be a more important factor than SES in terms of fertility and pregnancy outcomes. However, the influence of SES on female fertility should not be ignored. Socioeconomic status reflects an individual’s role at work and contribution to society, which may affect her life and health. First, women with high SES generally have more work stress and responsibilities, which means more working hours, long hours of work stress, work-related travel, and irregular lifestyles. These factors can negatively affect a woman’s menstrual cycle, reproductive hormone levels, and even ovulation, increasing the risk of infertility [40, 41]. Second, women of high SES may require more time and energy to pursue career advancement, leading them to neglect their health and fertility plans. Furthermore, women of high SES are often in competitive and stressful environments, which can negatively affect their physical and mental health, affecting fertility [42, 43]. Kim et al. further reported that the effect of work-life conflict on mental health is greater in higher-income groups than in lower-income groups [44].

Current research has paid more attention to the availability of medical resources for women with low SES while ignoring that women with high SES may be associated with a higher risk of infertility. Overall, there was some clinical value in our study. First, our study is a large cross-sectional study of women in the national American community to detect an interaction of age on the relationship between SES and infertility risk. Second, stratified multivariate logistic regression and interactive tests were used to explore the relationship between PIR and infertility risk. To support the robustness of our findings, we also performed a series of sensitivity analyses, which offered more credible clinical evidence than previous studies. Third, our findings will help understand the effect of different SES on infertility and improve the clinical practice of infertility treatment in terms of social factors. The government and all sectors of society should also consider the high SES population while helping disadvantaged groups with socioeconomic status. It is imperative to educate the public about the risk of various age-related reproductive complications so prospective parents can make informed decisions about when to start having children. The government should formulate relevant policies to help women balance work and childbirth, and improving the employment environment will be the direction of future efforts.

There are some limitations to this study. First, the study was conducted on women in the US; therefore, the findings do not apply outside the US. Exploring the relationship between SES and infertility across different countries and ethnicities will be the direction of future research. Second, the data were cross-sectional and observational, so causality cannot be established. Third, we relied on self-reports to assess the presence or absence of infertility, so recall bias was possible. Finally, treatment-related information on participants with infertility was not available.

## Conclusions

In conclusion, our study found that age may influence the association between PIR and infertility. A positive association was found between PIR and infertility risk in women aged 35 years or older, but no such association was found in women who were < 35 years old. This research will help increase awareness of the adverse effects of aging and poor employment environment on reproduction among the public and health care providers. Women, especially those with high SES, should make fertility plans in advance to reduce the adverse effects of delayed pregnancy. Women must know how to strike a balance of work and life with the pace of life accelerating. In the future, it is hoped that more safe and effective treatment strategies will enable those infertile patients to achieve parenthood as soon as possible. It is vital to continue exploring the relationship between SES and infertility across different countries and ethnicities which can potentially impact the outcomes in different populations.

### Electronic supplementary material

Below is the link to the electronic supplementary material.

Supplementary Material 1

## Data Availability

All the data sets we used for this study are publicly available from the NHANES website: https://www.cdc.gov/nchs/nhanes/index.htm. Any further inquiries can be directed to the corresponding author.

## Abbreviations

BMI: Body mass index
CI: confidence interval
NHANES: National Health and Nutrition Examination Survey
OR: odds ratio
PIR: poverty income ratio
SES: socioeconomic status
UK: United Kingdom
US: United States
VIF: variance inflation factor
WHO: World Health Organization
