# Supplementary material for: Age affects the association between socioeconomic status and infertility: a cross-sectional study
Source: BMC Womens Health. 2023 Dec 19;23:675. doi: 10.1186/s12905-023-02680-x (PMC10729442; doi:10.1186/s12905-023-02680-x)
Supplement: Supplementary file 1 — Supplementary Material 1 [file 12905_2023_2680_MOESM1_ESM.docx]

Supplementary Table 1 Relationship between all covariates and risk of infertility

| Covariates | OR_95CI | P-value |
| --- | --- | --- |
| Age | 1.04 (1.02~1.05) | <0.001 |
| Other Hispanic | 1.07 (0.68~1.67) | 0.769 |
| Non-Hispanic White | 1.47 (1.06~2.03) | 0.021 |
| Non-Hispanic Black | 1.18 (0.82~1.69) | 0.377 |
| Other Race | 1.05 (0.71~1.53) | 0.821 |
| Marital status(live alone) | 0.52 (0.41~0.65) | <0.001 |
| Education（High school） | 1.1 (0.76~1.59) | 0.624 |
| Education（More than high school) | 1.13 (0.83~1.55) | 0.43 |
| Health insurance | 0.93 (0.73~1.2) | 0.594 |
| Previous pregnancy | 1.99 (1.5~2.65) | <0.001 |
| BMI | 1.03 (1.02~1.04) | <0.001 |
| General health condition(Very good) | 1.71 (1.06~2.75) | 0.028 |
| General health condition(Good) | 1.86 (1.17~2.95) | 0.009 |
| General health condition(Fair) | 2.12 (1.3~3.47) | 0.003 |
| General health condition(Poor) | 2.3 (1.09~4.89) | 0.03 |
| Smoking | 1.34 (1.07~1.67) | 0.009 |
| Pelvic inflammatory disease | 1.9 (1.28~2.83) | 0.002 |
| Diabetes Mellitus | 2.16 (1.47~3.18) | <0.001 |
| Hypertension | 1.7 (1.33~2.19) | <0.001 |
| Coronary heart disease | 5.44 (1.21~24.38) | 0.027 |
| Cancer | 2.25 (1.4~3.63) | 0.001 |
| Stroke | 1.39 (0.53~3.65) | 0.5 |
| Vigorous recreational activities | 0.63 (0.49~0.81) | <0.001 |
| Moderate recreational activities | 0.9 (0.73~1.11) | 0.31 |
